# Evaluation of Quality of Nitrite-Free Fermented Roe Deer (*Capreolus capreolus*) Sausage with Addition of Ascorbic Acid and Reduced NaCl

**DOI:** 10.3390/foods13233823

**Published:** 2024-11-27

**Authors:** Karolina M. Wójciak, Paulina Kęska, Miroslava Kačániová, Natália Čmiková, Elżbieta Solska, Agata Ogórek

**Affiliations:** 1 Department of Animal Food Technology, Faculty of Food Science and Biotechnology, University of Life Sciences in Lublin, Skromna 8, 20-704 Lublin, Poland; karolina.wojciak@up.lublin.pl (K.M.W.); elzbieta.solska@up.lublin.pl (E.S.);; 2 Institute of Horticulture, Faculty of Horticulture and Landscape Engineering, Slovak University of Agriculture, Tr. A. Hlinku 2, 94976 Nitra, Slovakia; m.kacaniova@vizja.pl (M.K.); n.cmikova@gmail.com (N.Č.); 3 School of Medical & Health Sciences, University of Economics and Human Sciences in Warsaw, Okopowa 59, 01-043 Warszawa, Poland

**Keywords:** venison, roe deer, fermented sausage, TBARS, meat color, microbial analysis

## Abstract

This study aimed to investigate the possibility of producing fermented roe deer sausages using acid whey without the addition of sodium nitrite. Additionally, ascorbic acid was added to improve the oxidative stability of the product, and sodium chloride (NaCl) was partially replaced by potassium chloride (KCl) (7:3). The sausages were analyzed after fermentation (on day 30) and during post-production aging (i.e., 60 and 90 days after production at 4 °C) for their pH, water activity (a_w_), redox potential (ORP), thiobarbituric acid value (TBARS), and color parameters (CIE L*, a*, and b*). The microbiological status of the products was also profiled. During aging, the a_w_ and pH values were significantly lower (*p* < 0.05) in the variant with the addition of ascorbic acid. In all samples with the addition of acid whey, an increase in the TBARS value compared to the variant with sodium nitrite was observed, but among them, the variant with the substitution of NaCl by KCl was characterized by the lowest intensity of lipid oxidation. During post-production aging, the effect of acid whey on the loss of redness (a*) of the roe deer sausages was confirmed, with the lowest a* observed in samples with the addition of ascorbic acid. A total of 281 and 219 isolates with high scores were identified in the fermented deer sausages after fermentation (30 days) and storage (90 days), respectively. The most frequently isolated species from the fermented roe deer sausages were from the *Latilactobacillu* genus (*Latilactobacillus curvatus, Lati-lactobacillus sakei* subsp. *carnosus)* and *Leuconostoc* genus (*Leuconostoc mesenteroides*, *L. mesenteroides* subsp. *dextrani-cum*, and *Leuconostoc mesenteroides subsp. mesenteroides*).

## 1. Introduction

The vast majority of meat products contain nitrites (NO^2−^) and nitrates (NO^3−^), which are permitted in the European Union by Commission Regulation (EU) No. 1129/2011. Their use in meat products has positive attributes, including color fixation, flavor enhancement, antioxidant activity and, most importantly, preventing the risk of the bacterial contamination of cured meat, especially from *Clostridium botulinum*. However, despite these desirable technological effects, their use in meat products has been associated with the formation of potentially carcinogenic *N*-nitrosamines and the occurrence of a blood disease, i.e., methemoglobin. Therefore, there is an increasing demand among meat consumers for healthier products with good sensory properties. In recent years, actions have been proposed to limit the supply of nitrites in meat products [1,2] or to completely eliminate them [3,4]. In particular, much attention in the literature has been devoted to nitrite-free fermented meat products from pork [1], beef [4], lamb [5], and chicken [6] using acid whey. This is a by-product of the dairy industry, which, when applied to meat, can contribute to improving its quality without other quality losses typical of products with NO^2−^. Studies have shown that adding acid whey to meat mass can improve its moisture and texture, as well as affect the microbiological stability of the product. Acid whey naturally contains lactic acid, which is one of the main products of lactic fermentation. Microorganisms responsible for meat fermentation can use the favorable conditions of the reduced pH of the meat mass, resulting from the presence of lactic acid from whey, to increase the number of lactic acid bacteria in the product and accelerate the fermentation process of the product during maturation, especially in its early stages. In this context, whey can act as a natural preservative, supporting the development of desirable bacterial cultures and limiting the development of pathogens. This is particularly important in the case of game meat, where there is a greater risk of the microbiological contamination of the raw material compared to meat from farm animals such as pork or beef [1,2,3,4,5,6].

The rise in consumer interest in organic food is driven by improvements in healthy living choices. Food produced using certified organic farming methods, which is high in nutrients and free of preservatives or other functional additives, is becoming increasingly popular. Consequently, roe deer and other types of game meat can also meet consumer expectations. Due to their lipid content and composition, the consumption of game meat (especially that of wild ruminants) can be advantageous for human health [7,8,9,10,11]. Game meat is perceived as healthy, mainly due to its low fat content and organic farming methods (free-ranging animals). Additionally, the method for obtaining game is viewed by consumers as more environmentally and animal-friendly compared to that used for slaughter animals [12]. The literature on this subject reports that the factor limiting the production potential of roe deer is that the meat may be less attractive to consumers, i.e., it is harder and darker than the popular pork or beef [13]. However, satisfactory results can be achieved by appropriately designing processing technology. Moreover, research suggests that the unpleasant “gamey taste” is reduced in fermented game products [14]. In studies focusing on the production of fermented products using game meat, blesbok (*Damaliscus dorcas phillipsi*), springbok (*Antidorcas marsupialis*) [15], gemsbok (*Oryx gazella*), kudu (*Tragelaphus strepsiceros*), zebra (*Equus burchelli*), [16], wild boar (*Sus scrofa*), deer (*Cervus elaphus*) [17], and fallow deer (*Dama dama*) [4] have been used. However, there are few reports in the literature concerning products with a limited nitrite addition. Therefore, this could be seen as an opportunity for the meat industry’s development, with unconventional meat sources such as roe deer playing an increasing role in the production of functional meat products.

There is a need to increase the knowledge available to producers, which is necessary to make key decisions in the context of game meat processing. Due to the way this meat is obtained, particular attention is drawn to the associated microbiological risks. This information is important in the context of the trend in limiting the use of nitrites with strong antibacterial properties in meat products. The strategy proposed in this paper is related to the production of nitrite-free sausages, but with the addition of acid whey. Additionally, the impact of the addition of ascorbic acid and the possibility of limiting the use of NaCl (which was partially replaced by KCl) on the quality of roe deer sausages during fermentation and post-production aging are also considered. Ascorbic acid and sodium ascorbate act as antioxidants. Moreover, their reducing effects include the acceleration of nitric oxide formation, which can bind to myoglobin and reduce the *N*-nitrosamine content in cured meat products [18]. In turn, attempts to replace NaCl by using other chloride salts are primarily related to the increasing consumer awareness of sodium consumption and health. This is particularly important in the context of meat products undergoing fermentation and aging, in which the salting stage is a crucial step in securing the final quality of the product, where the use of salt is at a much higher level than that in the case of other meat products. Potassium chloride (KCl) is one of the most common substitutes proposed due to its similar molecular composition. However, it is impossible to completely replace NaCl with KCl. The main obstacle is the negative sensory characteristics of such products, as reported by other researchers. Horita et al. [19] suggest that replacing 50% of sodium chloride with potassium chloride allows for obtaining a stable meat emulsion due to the participation of chloride anions in the extraction of myofibrillar proteins. Nevertheless, it has been shown that this amount of substitution also leads to a reduction in sensory attributes and overall consumer acceptance [19]. In total, 33% more sodium chloride than potassium chloride was required to obtain a positive sensory experience in a model aqueous solution [20] and in dry-cured meat products [21]. Therefore, a 30% substitution of NaCl with KCl was used in the present study. Moreover, replacing NaCl with KCl on a molar basis provides a similar antimicrobial effectiveness against several pathogens, including *Aeromonas hydrophila*, *Enterococcus sakazakii*, *Shigella flexneri*, *Yersinia enterocolitica*, and three strains of *Staphylococcus aureus* [22], which may have a positive effect on microbiological safety in nitrite-free meat products.

In this study, a hypothesis that it is possible to produce high-quality, nitrite-free fermented roe deer sausages was adopted. The possibility of limiting the supply of sodium chloride in the product while fortifying it with sodium ascorbate to enhance its antioxidant potential was also considered. The quality determinants considered were the level of secondary fat oxidation products (TBARS), pathogenic microorganism levels, and color characteristics assessed using an instrumental method (ClE L*, a*, and b*). The proximate composition and physicochemical characteristics (pH, water activity, and oxidation–reduction potential (ORP)) were also assessed for a better interpretation of the results.

## 2. Materials and Methods

### 2.1. Meat Product Preparation

Roe deer meat and beef fat were purchased from a meat producer 48 h after slaughter. Ham muscle (85%) was first minced with a knife and cooled at 4 °C. Beef fat (15%) was also minced using a knife. The roe deer meat was divided into four equal samples. A curing mixture (99.4–99.5% sodium chloride and 0.5–0.6% sodium nitrite) was added to one of the stuffings in a proportion of 2.8% meat (control sample—C, Table 1). Acid whey and NaCl (S1; S2) or a mixture of NaCl:KCl (7:3) (S3; S4) were added to the remaining samples in a proportion of 2.8% of meat (Table 1). Ascorbic acid was also added to the selected variants (S2; S4) as an antioxidant in a proportion of 0.05% (Table 1). The experimental samples (S1–S4) did not contain any nitrites. Each meat sample was then minced separately using a universal machine type KU2-3EK (MESKO-AGD, Skarżysko-Kamienna, Poland) with 8 mm discs and mixed with ground backfat.

**Figure 1:**
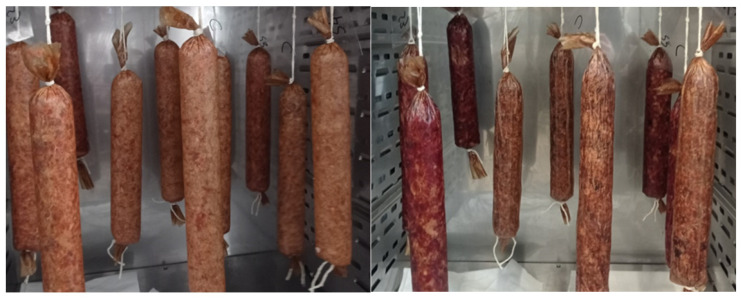
Roe deer sausages after meat product preparation (1 day; **left**) and after fermentation stage (30 days; **right**).

Five variants of sausage stuffings were prepared (Table 1), including stuffing with the curing mixture added (C), with acid whey and NaCl (S1), with acid whey, NaCl, and ascorbic acid (S2), with acid whey and NaCl:KCl (S3), and with acid whey, NaCl:KCl, and ascorbic acid (S4). The mixes were stuffed into casings (65 mm, VISCASE, Chicago, IL, USA) (Figure 1). In the next step, all variants were placed in fermentation chambers for 30 days under controlled humidity and temperature conditions (step I: 21 ± 1 °C; 58 ± 5 relative humidity, 3 days; step II: 15 ± 1 °C; 72 ± 4 relative humidity, 3 days; and step III: 13 ± 1 °C; 76 ± 1 relative humidity, 24 days). The finished products were then vacuum-packed in nylon-polyethylene bags and stored at 4 °C for further post-production aging.

### 2.2. Proximate Composition

The total protein content was assessed using the Kjeldahl method in accordance with the PN-75/A-04018 standard [23]. The analysis used an automatic Foss KjeltecTM8100 system (FOSS Analytical AB, Höganäs, Sweden). The protein level was determined as the product of the total nitrogen content multiplied by the factor 6.25. The result is expressed as a percentage. The total nitrogen content was determined by burning the research material (1 g) in Kjeldahl tubes at 420 °C for 60 min and distilling the samples by 5 min, using a stream of water (80 mL/sample) and 1M NaOH (50 mL). The total nitrogen content was determined according to the distillation apparatus manufacturer’s instructions. The total fat content was measured using the Soxhlet method [24]. The samples (after prior drying) were placed in a blotting paper thimble and placed in a wide tube of the Soxlet apparatus. The process was carried out for 2.5 h in the presence of a solvent—chloroform, at a temperature of 40 °C. After the process, the thimble was removed, the remaining chloroform was evaporated (dried in a dryer), and the thimble with its contents after the process was weighed. The fat content in percentage was calculated from the difference in the thimble weight before and after the process. The water content was determined according to ISO 1442:1997 [25]. For this purpose, the sample (3 g) was dried in a dryer at 103 °C for 6 h. After this time, the samples were taken out and cooled in a desiccator. The water content in percentage was calculated from the difference in the samples’ weight before and after the process. A proximate chemical composition evaluation was performed after fermentation (30 days).

### 2.3. The Physicochemical Parameters (a_w_, pH, Oxidation–Reduction Potential)

Water activity (a_w_) measurements were performed in a LabMaster aw (Novasina AG, Lachen, Switzerland) with a temperature-controlled measurement chamber (20 ± 1 °C). Before analysis, the device was calibrated using Novasina humidity standards. The water content was analyzed in a sample of freshly ground meat, placed directly in the measuring chamber of the apparatus.

The pH value was also assessed. For this purpose, a sample (10 g) was crushed and homogenized for 1 min with 90 cm^3^ of cold distilled water. The obtained homogenate was filtered through filter paper. The pH meter was calibrated before use against standardized buffer solutions at pH 4.00, 7.00, and 9.00 (0.05).

The oxidation–reduction potential (ORP) was determined using a complex platinum electrode type ERPt13 using a CPC-501 digital pH-conductometer (Hotek Technologies, Yelm, WA, USA). The determination was carried out in purified meat homogenate (10 g) in cold distilled water (90 mL). The obtained measurement result was converted into the value of the redox potential relative to the standard hydrogen electrode (at 20 °C). The result is expressed in millivolts (mV).

### 2.4. Content of Substances Reactive with Thiobarbituric Acid

The level of thiobarbituric acid reactive substances (TBARS) was performed according to the method described by Pikul et al. [26]. The TBARS index was determined in the filtrate obtained from the homogenate of 5 g of minced meat, 20 mL of cold perchloric acid (4%), and 400 μL of an alcoholic solution of butylhydroxytoluene. Up to 2.5 mL of an aqueous solution of 2-thiobarbituric acid (TBA reagent) was added to the filtrate and heated in a water bath (100 °C) for 20 min. After cooling to room temperature, the absorbance was measured at a wavelength of 532 nm using a Nicolet Evolution 300 spectrophotometer (Thermo Electron Corp., Waltham, MA, USA) against a reference sample containing 2.5 mL of a 4% perchloric acid solution and 2.5 mL of TBA reagent. The analysis used the spectrophotometric method of absorbance measurement at a wavelength of 532 nm, according to the following equation:

TBARS = 5.5 × Absorbance(1)

The results are expressed as mg of malondialdehyde (MDA) per kg of products (MDA/kg).

### 2.5. Color Measurement

The instrumental assessment of the meat color parameters was based on the measurement of sample reflectance in the CIE L*a*b* system, where L* means lightness, a* is the color ranging from green to red, and b* covers the color range from blue to yellow. The variants were kept at room temperature for 20 min to equilibrate the temperature, and 10 mm thick samples were then cut from the fermented sausages for measurements. X-Rite white and black standards were used for pre-measurement calibration. D65 illumination (daylight, 6500 K) and a colorimetric observer with a field of view of 10° were used for the tests. The instrumental conditions were a 12 mm diameter area aperture. The measurement was carried out in the range from 360 to 740 nm using an X-Rite spherical spectrophotometer (X-Rite, Inc., Grand Rapids, MI, USA).

### 2.6. Microbiological Analysis

Five-gram samples of sausages were combined with 45 mL of sterile saline (0.1%) in an Erlenmeyer flask and incubated in a shaking incubator (GFL 3031, Burgwedel, Germany) for 40 min. Then, the mixture was applied to a Petri dish and incubated under microorganism-dependent conditions. Thus, coliforms were detected using Violet Red Bile Lactose Agar (VRBL, Oxoid, Basingstoke, UK) after incubation at 37 °C for 24–48 h. Total viable counts (TVCs) were estimated after incubation for 48–72 h at 30 °C using Plate Count Agar (PCA; Oxoid, Basingstoke, UK). Lactic acid bacteria were cultured on De Man Rogosa and Sharpe Agar (MRS; Oxoid, Basingstoke, UK) for 48–72 h at 37 °C. Each test and analysis were performed in triplicate. Additionally, eight colonies per Petri dish were cultured on Tryptone Soya Agar (TSA; Oxoid, Basingstoke, UK) at 30 °C as part of a short reinoculation.

#### 2.6.1. Microorganism Identification Using Mass Spectrometry

Using reference libraries and the MALDI-TOF MS Biotyper (Bruker, Daltonics, Bremen, Germany), the microorganisms isolated from the deer meat samples were identified.

#### 2.6.2. MALDI-TOF Biotyper Matrix Solution

An initial stock solution (the MALDI-TOF matrix solution) was organically modified. The standard solution contained 2.5% trifluoroacetic acid, 47.5% water, and 50% acetonitrile. To prepare 1 mL of the stock solution, 475 µL of filtered water, 25 µL of pure 10% trifluoroacetic acid, and 500 µL of pure 100% acetonitrile were combined. The organic solvent was mixed with the “HCCA matrix portioned” in a 250 mL Eppendorf flask. All matrix components were supplied by Aloqence in Vrable, Slovakia.

#### 2.6.3. Microbial Identification

The sample preparation process followed earlier guidelines, which required dividing the Petri plate into eight separate colonies. After transferring the biological material from the Petri plate to an Eppendorf tube with 300 µL of distilled water, 900 µL of ethanol was added, and the mixture was thoroughly mixed. The resulting mixture was centrifuged for two minutes at 10,000× *g* using a ROTOFIX 32A (Ites, Vranov, Slovakia). After removing the supernatant, the precipitate was extracted from the Eppendorf tube and allowed to air dry at room temperature (20 °C). Subsequently, the particles were mixed with 30 μL of acetonitrile and 30 µL of 70% formic acid and centrifuged at 10,000× *g* for 2 min. One microliter of the liquid mixture and 1 mL of the MALDI matrix solution were added to a MALDI plate and dried. The microorganisms were identified using a MALDI-TOF mass spectrometer (Bruker, Daltonics, Bremen, Germany). A Microflex LT MALDI-TOF mass spectrometer (Bruker Daltonics, Bremen, Germany) automatically created mass spectra in the linear positive mode, covering a mass range of 2000–20,000 Da. The Bruker bacterial test standard was used to calibrate the apparatus. The obtained mass spectra were assessed using the MALDI Biotyper 3.0 tool (Bruker Daltonics, Bremen, Germany). The identification scores were interpreted as follows: a score of less than 1.700 was deemed as unreliable for identification; a score from 1.700 to 1.999 indicated probable genus identification; a score from 2.000 to 2.299 suggested genus identification with probable species identification; and a score from 2.300 to 3.000 indicated highly probable species identification.

### 2.7. Statistical Analysis

The study was randomized and repeated three times, and every sample was measured in triplicate. The analyses were performed in nine repetitions. The values of various parameters are expressed as mean ± standard deviation. The data were subjected to one-way or two-way analysis of variance (ANOVA) and Tukey’s comparison of means test (*p* < 0.05). The Pearson correlation coefficient (r) between selected predictors (qualitative features) was determined and is presented as a heat map. Multivariate analyses (cluster analysis, CA) were also carried out. The data set was treated using Ward’s method and combined with the square of the Euclidean distance as a measure of similarity for multivariate analysis. For cluster analysis, a hierarchical method was used, which included measuring the similarity between objects (variants), and samples with maximum similarities were grouped graphically on dendrograms. The acquired data were processed using the Statistica^®^ 13.1. (StatSoft, Kraków, Poland) analytic software.

## 3. Results and Discussion

### 3.1. Assessment of Proximate Composition

**Figure 2:**
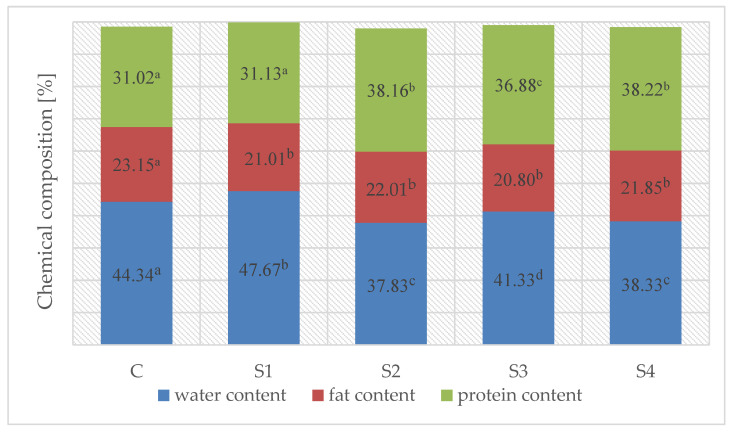
The percentage contents of water [%], fat [%], and protein from roe deer sausages. [^a–d^—values marked with different lower case letters differ significantly (*p* < 0.05) in the same analysis; C—control sample with sodium nitrite, S1—sample with acid whey and 2.8% NaCl, S2—sample with acid whey, 2.8% NaCl and 0.05% ascorbic acid, S3—sample with acid whey and 2.8% NaCl:KCl (7:3), and S4—sample with acid whey, 2.8% NaCl:KCl (7:3) and 0.05% ascorbic acid].

The results of assessing the water, fat, and protein contents in the test variants after fermentation are presented in Figure 2. The lowest differences in content between individual variants of the fermented sausages were determined for fat. The highest fat content was recorded for the control sample (C)—23.15% (*p* < 0.05), while lower values were noted for the nitrite-free samples (20.80–22.01%) (*p* > 0.05). Variants with the addition of ascorbic acid (S2 with the addition of NaCl and S4 with the addition of NaCl:KCl (7:3)) were characterized by the lowest water content and the highest protein content (*p* < 0.05).

In the production of the fermented sausages, two key stages (fermentation and aging) that modified the contents of the main meat components (carbohydrates, proteins, and lipids) can be distinguished. Changes in the basic chemical composition of the meat after the fermentation and maturation process occurred naturally. They were primarily the result of water loss from the product due to the interaction of the temperature and humidity conditions in the fermentation chamber. The research findings suggest that the use of ascorbic acid may have led to an increased water loss from the product, reaching an average level of 38.08% in the S2 and S4 variants. The presence of ascorbic acid caused a decrease in the pH of the meat matrix, rich in proteins. When this pH value of proteins reached a value equal to their isoelectric point, proteins had the lowest water holding capacity, which explains the increase in water loss from the product. In contrast, in the control sample (C), the water content was 44.34%. This would result in a change in the proportions of the remaining meat components (fat and protein), with the protein fraction being more sensitive (*p* < 0.05). The protein content in all variants ranged from 31.02% to 38.22%. Paleari et al. [27] recorded the protein content at 44.6% in dry-cured deer products after 30 days of aging. In turn, Ranucci et al. [28] reported a protein content of 29% in salami with 50% roe deer meat and 50% pork meat. This effect after the drying process (which occurs simultaneously with fermentation) may be influenced by the reduction in NaCl content, which affects the solubility and binding capacity of proteins [29]. Ibanez et al. [30] found that reducing the NaCl ratio affected the ability of proteins to retain water and the drying process. Zhang et al. [31] suggested that the mechanism of replacing NaCl with KCl influenced the water retention process in salted pork. The authors also showed that the water holding capacity in samples containing K+ also increased [31]. Di Luccia et al. [32] and Zhang et al. [31] also indicated that K+ ions may increase the rate of protein degradation due to oxidation, which, in turn, may increase the net negative charge on the surface of myofilaments in muscle tissues and affect the water holding capacity [33]. These results do not correspond to the results presented in the current study, as a lower water content was observed in samples of fermented roe deer with 30% KCl instead of NaCl. Moreover, as shown, the effect of technological treatments applied on the water content in the product was enhanced by the use of ascorbic acid, which indirectly accelerated the evaporation of water from the product. It is worth noting that the gradual loss of water from the product during the drying and aging stages led to less water being available for microorganisms to grow and survive. The addition of ascorbic acid in the production of nitrite-free roe deer sausages may contribute to a greater microbiological safety of products due to an increased water loss.

### 3.2. Assessment of Physicochemical Parameters (pH, a_w_, ORP)

The intensification of biochemical changes during the production of aging meat products can be monitored by changes in the basic physicochemical parameters of the products. In the case of fermented meat products, pH and a_w_ are very important, because they will influence the course of chemical and enzymatic reactions and the growth of microorganisms during production. In addition, the health and safety of fermented meat products are based on combined hurdle technologies, including pH and a_w_. Through the production process, efforts should be made to lower the pH and a_w_ to create an environment that disturbs the growth of bacteria that cause food spoilage. Fermented sausages have typically been divided according to their pH and water activity levels into (a) “very perishable” (pH > 5.2; a_w_ > 0.95), (b) “perishable” (pH 5.2–5.0 or a_w_ = 0.95–0.91), or (c) “shelf-stable” (pH 5.2 and a_w_ < 0.95 or only pH < 5.0 or only a_w_ < 0.91) [34].

Table 2 presents the results of an analysis of the physicochemical parameters of fermented roe deer sausages. The influence of technological treatments and refrigerated storage time on the water activity and pH of the products was observed. However, the water activity of the aging roe deer sausages showed different trends, depending on the technological process used. Generally, there was an increase in a_w_ on day 60 of the analysis, followed by a decrease in the last research period (day 90) in the variants with sodium nitrite (C). A different tendency was observed in the case of samples with the addition of acid whey, i.e., a systematic decrease in a_w_ up to 90 days of storage for the S1 and S3 samples (*p* < 0.05) or the stability of the parameter in question until day 60 of the process, followed by a significant increase in the parameter in question on day 90 in the case of variants with the addition of ascorbic acid (S2 and S4; *p* < 0.05)). There were no statistically significant (*p* > 0.05) differences found in the water activity between the samples with a reduced amount of sodium and potassium chloride (S3) and the variant containing the addition of NaCl only (S1) until day 60 of the analysis. Moreover, Campagnol et al. [35] found no significant differences in a_w_ (*p* > 0.05) when a 50% substitution of NaCl with KCl was used in a fermented sausage recipe. However, Zampouni et al. [36] recorded higher a_w_ values after 14 days of fermentation in samples with a 50% substitution of NaCl with KCl. There was also an influence of the use of ascorbic acid on the a_w_ value, which was significantly lower (*p* < 0.05) in the samples with the addition of this acid throughout the entire experiment period, correlating with the lower water content (Figure 2) in these variants. A low aw value in the final product will contribute to an improved product safety. The water activity of all the samples at the end of the process was below 0.920, which is defined as the minimum value required for the growth of many undesirable microorganisms, including Pseudomonas (a_w_ > 0.97), Clostridium botulinum (a_w_ > 0.93 ÷ 0.96), Salmonella (a_w_ > 0.94), and Listeria monocytogenes (a_w_ > 0.92) [37].

Another parameter important for assessing the quality and health safety of fermented meat products is the pH value. The value of this coefficient was found to depend on the processing method and ripening time of the roe deer sausages. Generally, in the current study of fermented roe deer sausages, the initial pH ranged from 4.80 ± 0.01 (S4) to 5.10 ± 0.02 (S1) after fermentation (30 days) and dropped to values ranging from 4.64 ± 0.01 (S4) to 4.98 ± 0.01 (S1) after storage in refrigeration (4 °C; 90 days). Similar pH values were obtained by Karwowska and Dolatowski [38] in a fermented sausage with acid whey and without nitrites/nitrates produced from deer meat, where the pH values ranged from 4.59 (day 0) to 4.66 (day 90). It is worth emphasizing that such low pH values (<5.0) are an important factor inhibiting the development of pathogenic microorganisms. The technological procedure consisting of replacing part of NaCl with KCl in the process of preparing fermented game sausage did not contribute (*p* > 0.05) to a change in the pH value of the products (except for the S1 and S3 samples on day 30 of analysis, *p* < 0.05). Immediately after fermentation (day 30), no significant (*p* > 0.05) differences were observed between the control sample (C) and the nitrite-free variants (with acid whey), including the S2 (NaCl) and S4 (NaCl:KCl) variants. Kononiuk and Karwowska [4] also found no effect of acid whey on the pH value of fermented fallow deer sausages. However, during subsequent stages of sausage aging in a refrigerator, a systematic decrease (*p* < 0.05) in the value of the discussed parameter was observed in the variants with the addition of acid whey compared to the control variant (with the addition of sodium nitrite). Moreover, the tendency observed in the studies concerning a decrease in the pH of samples during refrigerated storage was enhanced by the use of ascorbic acid. Variants with the addition of ascorbic acid from the first research period (day 30) were characterized by the lowest pH values (*p* < 0.05) (S2 and S4 vs. S1 and S3), and this relationship persisted until day 90 of aging.

As can be seen from Table 2, the ORP level of the test variants increased between day 30 and day 60 of the analysis and then stabilized on day 90 of cold storage (C, S1, S2, and S3) or decreased, as in the S4 variant with a reduced sodium chloride content and the addition of ascorbic acid. Considering the effect of the addition of potassium chloride to limit the supply of NaCl, significantly lower ORP values were recorded (S1 vs. S3) after 30 days of refrigerated storage, while in further research periods, the effect of the process used was insignificant (*p* > 0.05). The addition of ascorbic acid (S2 and S4) variants was associated with a significantly higher level of ORP on day 60 of analysis, which had no effect in the remaining study periods.

### 3.3. Assessment of Lipid Oxidation Degree Based on TBARS Value

**Figure 3:**
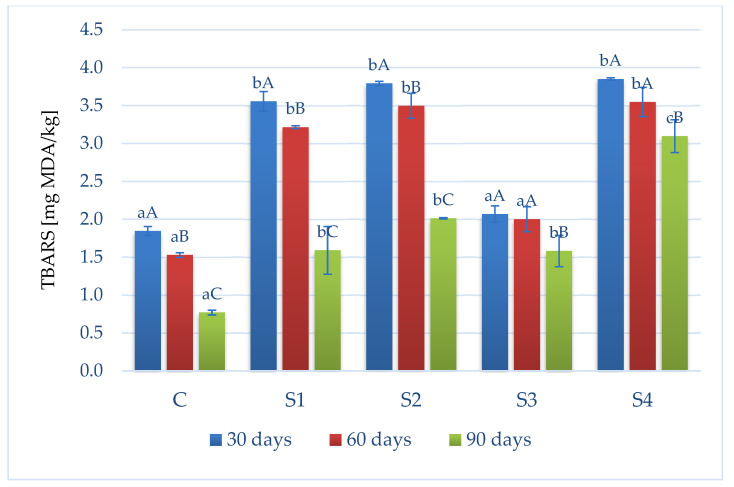
TBARS values in fermented roe deer products during cold storage (4 °C, 90 days) [^A–C^—values marked with different capital letters are significantly different (*p* < 0.05) in the same sample; ^a–c^—mean values marked with different lowercase letters are significantly different (*p* < 0.05) at the same time. The results are presented as means ± standard deviation C—control sample with sodium nitrite, S1—sample with acid whey and 2.8 % NaCl, S2—sample with acid whey, 2.8 % NaCl, and 0.05 % ascorbic acid, S3—sample with acid whey and 2.8% NaCl:KCl (7:3), S4—sample with acid whey, 2.8 % NaCl:KCl (7:3), and 0.05 % ascorbic acid].

Lipid oxidation can lead to the formation of hydroperoxides, which are chemically unstable and prone to cleavage, leading to secondary oxidation products such as aldehydes, ketones, and epoxides. Malondialdehyde (MDA) is the main secondary product of lipid oxidation and is widely assessed using the TBARS assay. The results of the presented tests are given in Figure 3. After fermentation (day 30), the lowest TBARS value of 1.85 mg MDA/kg was observed in the control samples with sodium nitrite (C). At this time, the remaining variants with the addition of acid whey (S1, S2, and S4) had significantly higher TBARS levels (*p* < 0.05). The exception was the variant with a reduced sodium chloride content (NaCl:KCl; S3), for which results similar to the levels typical of the control sample (C; day 30) were observed. Similar results were obtained by Karwowska et al. [4,39], indicating the absence of a limiting effect of the addition of acid whey on the lipid oxidation process in fermented sausages. During the entire refrigerated storage period, a decrease (*p* < 0.05) in the content of substances reactive with thiobarbituric acid (TBARS) was recorded for all analyzed variants. The use of ascorbic acid did not limit the fat peroxidation process in products with an addition of NaCl (without KCl). Higher TBARS values were observed on day 90 of analysis when using KCl (S4) as a substitute for this salt and adding ascorbic acid, among other samples. A characteristic feature of ascorbic acid is its reducing ability. In the presence of oxygen, ascorbic acid tends to oxidize with a strong effect, especially with respect to catalytic metals, removing environmental oxygen supplies. In addition, ascorbic acid can react with free radicals, stopping chain reactions. Balev et al. [40] reported the antioxidant effect of ascorbic acid on lipid oxidation in dry fermented sausages, but the addition of this ingredient in amounts below 200 ppm can convert ascorbic acid into a pro-oxidant agent [41]. The research results presented by Karwowska et al. [42] indicated the synergistic effect of whey and ascorbic acid regarding the reduction in lipid oxidation products in nitrite-free beef sausage. This was not confirmed by the present study, where the differences between the S2 (with NaCl) and S3 (NaCl and the addition of ascorbic acid) research variants during storage were insignificant (*p* > 0.05). On the other hand, the results obtained in this study are consistent with those of Berardo et al. [43], who did not find a greater interaction between sodium nitrite and sodium ascorbate (sodium salt of ascorbic acid) compared to the separate addition of sodium nitrite or sodium ascorbate on lipid oxidation in dry-fermented sausages. Moreover, analyzing the data presented in Figure 3, it can be concluded that the use of ascorbic acid and the simultaneous limitation of the addition of NaCl (S4) had a pro-oxidant effect during long storage in refrigeration conditions (day 60). On the other hand, a decrease in the TBARS value of fermented roe deer was observed when NaCl was partially replaced by KCl (S3; variants without the addition of ascorbic acid). This trend is consistent with reports by other authors, e.g., Cheng et al. [44] confirmed the reduction in lipid oxidation when salting pork meat with the addition of KCl. In general, data from the literature indicate that NaCl has a pro-oxidant effect on lipid oxidation, which can be attributed to its ability to disrupt the integrity of the cell membrane, facilitating the access of oxidizing agents to lipid substrates, releasing ions from iron-containing molecules, and inhibiting superoxide dismutase [45]. However, since KCl has a lower pro-oxidant effect than NaCl [46], its use may delay lipid oxidation [44,47], which can be observed especially up to day 60 of the aging of fermented roe deer sausages.

### 3.4. Evaluation of Color Parameters

The acceptability of products depends largely on their organoleptic properties, including visual impressions. Meat color is influenced by several endogenous factors, in particular pH, muscle fiber type, the presence of antioxidants and lipid oxidation, as well as mitochondrial activity in muscles [48]. Moreover, factors such as the diet of wild animals affect the color of meats [49]. In the first research period, e.g., 30 days after the production of the roe deer meat sausages, no statistically significant differences (*p* > 0.05) in the lightness of the products were observed (Table 3). In the next research period (day 60), the L* color parameters (lightness) increased compared to the previous period (30 days) in the control sample (C) and the S4 sample (with a reduced sodium chloride content and the addition of ascorbic acid). During this period of time (day 60), a decrease in the brightness of the products (L*) was also observed in the samples with the addition of NaCl (S1; S2) compared to the control sample (C) (*p* < 0.05), as well as the stabilization of this parameter in the variants with the addition of the NaCl:KCl mixture (S3; S4) (*p* > 0.05). At the end of the storage period (90 days), it was observed that the presence of ascorbic acid played a stabilizing role in the production of nitrite-free, fermented roe deer sausages. This was evident, as it led to the development of products with a lighter color (S2 and S4) and higher lightness value compared to the variant with sodium nitrite (C).

The addition of acid whey to the production of fermented roe deer sausages significantly reduced the redness parameter (a*) value compared to the control sample (C) (Table 3). The pigmentation enhancement in the sodium nitrite test was most likely caused by the formation of nitrosomyoglobin in the meat and the formation of a non-permeable pigment due to the absence of water (Figure 2). On the other hand, the use of whey led to the redness of the maturing fallow deer sausages [4]. The effect of substituting the Na+ ion with K+ on the discussed parameter was not confirmed. The use of ascorbic acid and a decreased NaCl addition by its partial replacement with KCl were associated with the redness of the fermented sausages on day 90 after fermentation (*p* < 0.05). The b* parameter value is visible with the yellow pigment originating from the oxidation reaction of lipids and amino acids in phospholipid head groups or amino acids in proteins [50]. Considering the b* parameter value (Table 3), a significantly higher value of this parameter was observed in samples with NaCl (S1 and S3) compared to the other test variants on day 60 of analysis. In the current experiment, the effect of reducing the sodium chloride content or adding ascorbic acid was also examined, but the effect on the b* parameter value of the fermented deer sausages during cold storage was not confirmed.

### 3.5. Microbiological Analysis

In this study, the count of coliform bacteria ranged from 1.28 log CFU/g in the fermented deer sausage samples with 50 mL/kg acid whey and 2.8% salt (S1) to 1.45 log CFU/g in the control samples (C). The total viable count in the fermented deer sausages after aging ranged from 3.10 log CFU/g in S2 to 3.84 log CFU/g in the control samples (C). Lactic acid bacteria ranged from 3.36 log CFU/g in the control group of roe deer sausage samples (C) to 3.67 log CFU/g in the roe deer sausage samples with 50 mL/kg acid whey and 2.8% NaCl:KCl (7:3) (S3) (Table 4).

**Figure 4:**
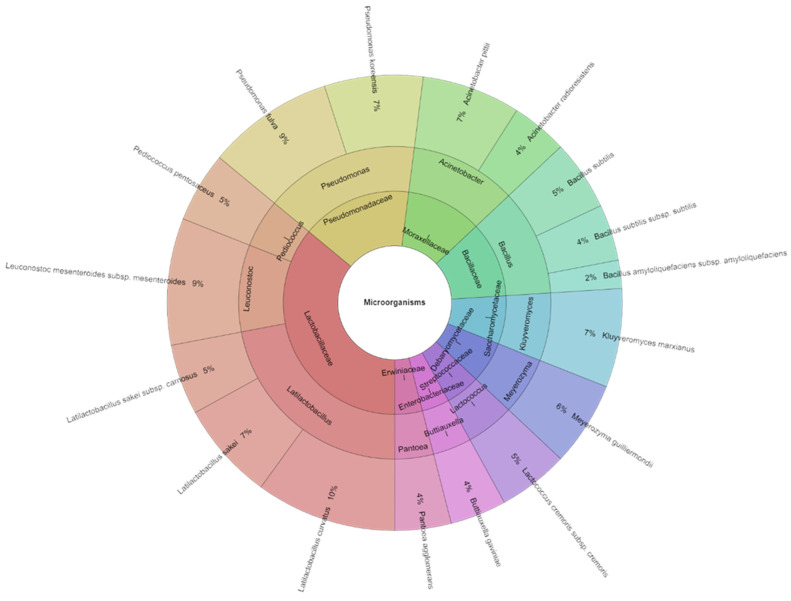
Krona chart: isolated species, genera, and families from fermented deer sausages after the fermentation stage (after 30 days of production).

Certain issues arise when roe deer is used to make fermented sausages. The typical qualities and characteristics of fermented sausages result from chemical, metabolic, physicochemical, and microbiological changes that occur during processing [51,52]. The nutrient composition of a product and the course of nutrient changes during processing can be significantly influenced by variations in the composition and characteristics of raw materials [53,54]. For this reason, it is justified to compare the technological and microbiological parameters used in producing organic fermented sausages from roe deer. A total of 281 isolates with high scores were obtained and identified in the fermented roe deer sausages after fermentation (30 days). These isolates belonged to 17 species, 11 genera, and 9 families. The most frequently isolated species from the fermented roe deer sausages were *Latilactobacillus curvatus*, which had a 10% presence, and *Leuconostoc mesenteroides subsp. mesenteroides* and *Pseudomonas fulva*, with a 9% presence (Figure 4).

The growth of certain bacteria, such as lactic acid bacteria, is desirable, as this regulates the proper progression of the fermentation process. Therefore, microbiological changes during the preparation of fermented meat are especially significant [4]. Conversely, the presence of pathogenic bacteria negatively affects the food safety of products. Increasing the population of lactic acid bacteria during the meat fermentation process is crucial, as these bacteria are primarily responsible for producing lactic acid and bacteriocins with the potential to serve as natural meat preservatives [55]. Despite this, sausages made from fallow deer had fewer Enterobacteriaceae. This could suggest that a decrease in the *Enterobacteriaceae* count is not solely attributable to an increase in the number of lactic acid bacteria. Several studies have extensively documented a correlation between LAB and the *Enterobacteriaceae* concentration during the preparation of dry-cured products [56]. Table 5 shows the microbiological quality of the fermented roe deer sausages after 90 days of production. The count of coliform bacteria varied from 1.66 log CFU/g in the fermented roe deer sausages with 50 mL/kg acid whey and 2.8% NaCl:KCl (7:3) (S3) to 2.38 log CFU/g in the control variants (C). The total viable count ranged from 3.36 log CFU/g in the fermented roe deer sausage samples with 50 mL/kg acid whey and 2.8% salt (S1) to 3.59 log CFU/g in S3 (with 50 mL/kg acid whey and 2.8% NaCl:KCl (7:3)). The lactic acid bacteria count ranged from 3.39 log CFU/g in the control variants (C) to 3.81 log CFU/g in the fermented roe deer sausages with 50 mL/kg acid whey, 2.8% NaCl:KCl (7:3), and 0.05% ascorbic acid (S4). There is a dearth of information on the microbiological quality of game meat. In their analysis of the initial bacterial contamination of fresh wild boar meat, Peruzy et al. [57] found counts that were significantly higher than those found in the current study. The average count of *Pseudomonas* spp. was 3.82 log CFU/g, the average count of *Lactobacillus* spp. was 3.65 log CFU/g, and the average TVC was 4.76 log CFU/g. The meat of wild boar kept for 21 days at 0 °C under aerobic conditions was examined by Borilová et al. [58]. In a different investigation, all groups of deer samples had Bacillus cereus, all control groups contained *Staphylococcus epidermidis* and *S. capitis*, and samples treated with 0.5% *Litsea cubeba* essential oils [EOs] contained *S. capitis*. From the control groups of samples, four species from the genus *Pseudomonas*—*Pseudomonas lundensis, Pseudomonas fragi*, *Pseudomonas taetrolens,* and *Pseudomonas corrugate*—were isolated. *Sphingomonas leidyi* and *S. paucimobilis* were identified in both the vacuum-packed control group and the aerobic control group. *S. leidyi* was isolated from deer meat samples treated with 0.5% *Litsea cubeba* EO and rapeseed oil (control). In a study of vacuum-packed products, *Brevibacillus borstelensis* was isolated from the aerobic control group and *Pantoea agglomerans* was isolated from the control group [59].

**Figure 5:**
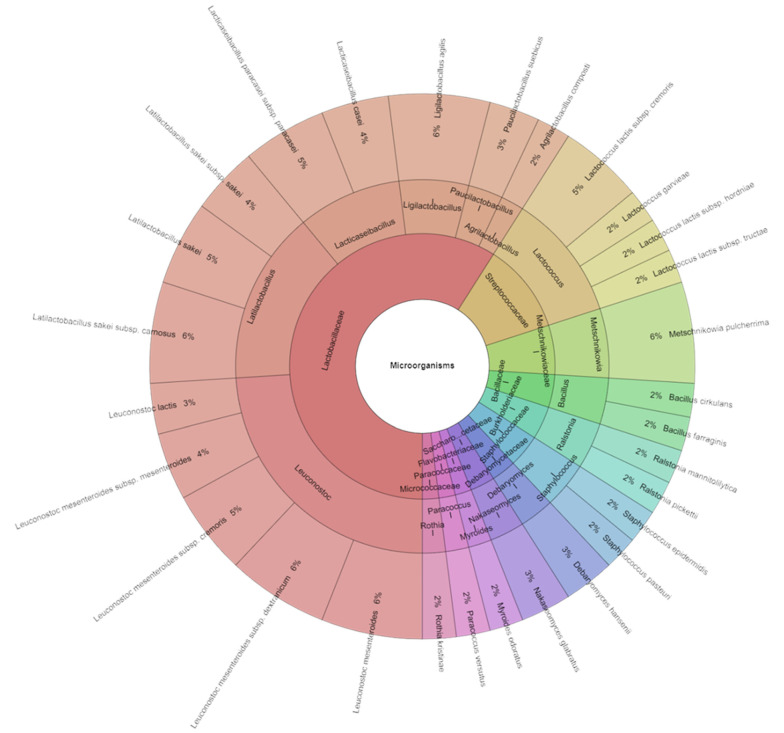
Krona chart: isolated species, genera, and families from fermented deer sausages after 90 days of production (4 °C).

After 90 days of storage, 291 isolates with a high score were identified in the fermented deer sausages. These isolates came from 11 families, 16 genera, and 29 species. After this time (90 days) of storage, the most frequently isolated species from the fermented deer sausages were *Latilactobacillus sakei* subsp. *carnosus*, *Leuconostoc mesenteroides*, *L. mesenteroides* subsp. *dextranicum*, *Ligilactobacillus agilis*, and *Metschnikowia pulcherrima*, with a 6% presence (Figure 5). There is not much knowledge available about the microbiota in game meat. *Pseudomonas, Pantoea*, *Escherichia*, and *Acinetobacter*, as well as a significantly frequent *Salmonella* [58], are the most commonly isolated families in wild boar meat. After analyzing samples of roe deer meat, Asakura et al. [60] identified the *Escherichia coli* serotype that produces the *Shiga* toxin in deer meat. In samples of wild boar meat, they also detected the coliform bacterium *E. coli*, as well as bacteria belonging to the genera *Acinetobacter* and *Arthrobacter.* Maksimovic et al. [61] identified coliform bacteria and a significant count of Bacillus cereus bacteria in deer sausage samples.

### 3.6. Statistical Support of Results

**Figure 6:**
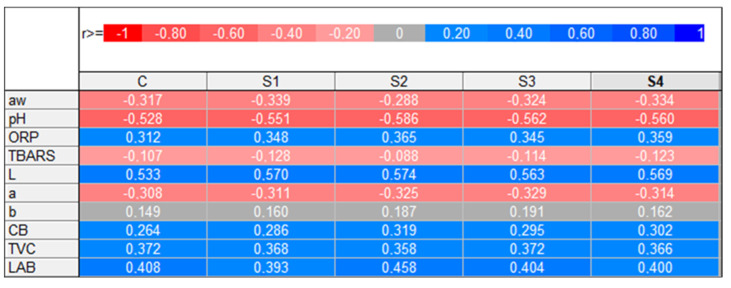
Linear correlation values (r) presented as a heat map.

The levels of the statistical significance (*p*-values) of the main effect (variant) and its influence on various chemical and physical parameters are presented in Table 6. In the case of attributes such as the ORP, lightness (L), and yellowness (b) of sausages and their microbiological state (CB, TCV, and LAB), no statistically significant interactions (*p* > 0.05) were noted. The applied technological treatment was a significant factor influencing the a_w_, pH, TBARS, and redness parameter (a) of the fermented venison sausages. The multiplicity of correlations (r) for two variables (variant vs. the evaluated parameter) is additionally presented in the form of a heat map (Figure 6).

**Figure 7:**
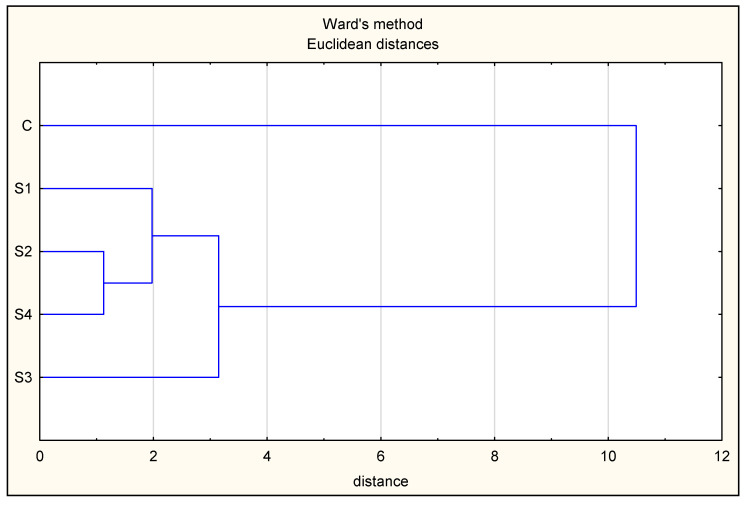
Dendrogram presenting the result of cluster analysis using the agglomeration method (the analysis included significantly differentiating factors (aw, pH, TBARS, and redness (a*))).

Figure 7 shows the clustering pattern for the variant (C, S1, S2, S3, and S4) data set. Variant C (with sodium nitrite) formed a distinct group, and the acid whey samples (S1–S4) were more closely related to each other, forming a distinct cluster. This second cluster formed primary subclusters, among which the S3 variant (with NaCl substitution by KCl) formed a distinct group due to its lower aw (Table 2) and lower TBARS index level (Figure 3) among all nitrite-free variants. Cluster analysis also clearly distinguished a secondary subcluster formed by the variants with added ascorbic acid (S2 and S4). This technological procedure was associated with a lower aw in the variants (Table 2), along with a faster water loss during fermentation, a lower pH (Table 2) of the product, and lower redness values (day 90; Table 3).

## 4. Conclusions

The test results confirmed the possibility of using only salt and acid whey to preserve roe deer meat. The fermented deer sausage was characterized by an appropriate degree of acidification (pH from 4.64 to 5.10) and water activity (from 0.712 to 0.835) favoring fermentation, which was confirmed by the results of microbiological analyses. The tested sausages were also characterized by a satisfactory microbiological quality and the absence of pathogenic bacteria. The most frequently isolated species from the fermented roe deer sausages were *Latilactobacillus curvatus*, *Leuconostoc mesenteroides* subsp. *mesenteroides*, and *Pseudomonas fulva*. Research has shown that a partial replacement of sodium chloride with potassium chloride (7:3) is possible in the case of fermented deer sausage technology without the deterioration of microbiological quality. Moreover, partially adding KCl (30%) to the salt mixture improved the oxidative stability and enhanced the reddish color of the fermented roe deer sausages.

## Figures and Tables

**Table 1:** Recipe for variants of roe deer sausages (per kg of stuffing).

Ingredient	Variant				
	C	S1	S2	S3	S4
Meat (game) (g)	850	850	850	850	850
Bovine backfat (g)	150	150	150	150	150
Ascorbic acid (g)	-	-	0.5	-	0.5
Water (mL)	50	-	-	-	-
Acid whey (mL)	-	50	50	50	50
Sodium nitrite (g)	0.14	-	-	-	-
NaCl (g)	27.86	28	28	-	-
NaCl:KCl (7:3) (g)	-	-	-	28	28
Glucose (g)	6	6	6	6	6

**Table 2:** Physicochemical parameters of fermented roe deer products during cold storage (4 °C, 90 days).

Time [Days]	Samples				
	C	S1	S2	S3	S4
Water activity (a_w_)					
30	0.829 ± 0.002 ^aA^	0.835 ± 0.001 ^bA^	0.713 ± 0.001 ^cA^	0.834 ± 0.001 ^bA^	0.729 ± 0.001 ^dA^
60	0.859 ± 0.007 ^aB^	0.821 ± 0.004 ^bB^	0.711 ± 0.001 ^cA^	0.823 ± 0.006 ^bA^	0.727 ± 0.001 ^dA^
90	0.831 ± 0.010 ^aA^	0.769 ± 0.005 ^bC^	0.735 ± 0.003 ^cB^	0.712 ± 0.001 ^dB^	0.813 ± 0.002 ^eB^
pH value					
30	5.01 ± 0.02 ^aA^	5.10 ± 0.02 ^bA^	4.81 ± 0.03 ^cA^	5.04 ± 0.02 ^aA^	4.80 ± 0.01 ^cA^
60	5.03 ± 0.20 ^aA^	4.96 ± 0.04 ^aB^	4.69 ± 0.02 ^bcB^	4.91 ± 0.03 ^abB^	4.65 ± 0.02 ^cB^
90	4.94 ± 0.06 ^aA^	4.98 ± 0.01 ^aB^	4.69 ± 0.04 ^bB^	4.88 ± 0.03 ^aB^	4.64 ± 0.03 ^bB^
Oxidation-reduction potential (ORP) (mV)					
30	334.17 ± 6.09 ^aA^	348.43 ± 1.80 ^bA^	348.97 ± 2.94 ^bA^	336.17 ± 6.21 ^aA^	334.17 ± 3.17 ^aA^
60	357.90 ± 2.80 ^aB^	359.57 ± 4.51 ^abB^	368.57 ± 2.63 ^cB^	366.77 ± 2.33 ^bcB^	377.83 ± 3.46 ^dB^
90	366.17 ± 2.25 ^aB^	358.97 ± 2.15 ^aB^	367.67 ± 1.45 ^aB^	356.90 ± 6.44 ^aB^	365.00 ± 6.32 ^aC^

^A–C^ The values marked with different capital letters are significantly different (*p* < 0.05) in the same sample; ^a–e^ mean values marked with different lowercase letters are significantly different (*p* < 0.05) at the same time. The results are presented as means ± standard deviation. C—control sample with sodium nitrite, S1—sample with acid whey and 2.8% NaCl, S2—sample with acid whey, 2.8% NaCl, and 0.05% ascorbic acid, S3—sample with acid whey and 2.8% NaCl:KCl (7:3), and S4—sample with acid whey, 2.8% NaCl:KCl (7:3), and 0.05% ascorbic acid.

**Table 3:** Color parameters of aging roe deer sausage during cold storage (4 °C, 90 days).

Time [Days]	Samples				
	C	S1	S2	S3	S4
Lightness (L*)					
30	41.82 ± 8.08 ^aA^	43.96 ± 5.56 ^aA^	44.09 ± 4.94 ^aA^	51.01 ± 1.58 ^aA^	48.34 ± 4.18 ^aA^
60	55.98 ± 3.82 ^aB^	50.42 ± 3.88 ^bA^	47.744 ± 1.85 ^bA^	51.13 ± 2.38 ^abA^	54.82 ± 1.96 ^abB^
90	53.19 ± 3.33 ^aB^	47.03 ± 1.49 ^bA^	55.04 ± 3.04 ^aB^	51.95 ± 1.72 ^aA^	53.60 ± 1.22 ^aB^
Redness (a*)					
30	7.33 ± 0.06 ^aA^	3.17 ± 0.36 ^bA^	3.66 ± 0.52 ^bA^	3.55 ± 0.48 ^bA^	3.46 ± 0.44 ^bA^
60	7.45 ± 0.56 ^aA^	3.11 ± 0.59 ^bA^	3.72 ± 0.40 ^bA^	3.67 ± 0.38 ^bA^	3.54 ± 0.38 ^bA^
90	7.71 ± 1.00 ^aA^	4.22 ± 0.59 ^bcA^	3.21 ± 0.55 ^cA^	4.46 ± 0.43 ^bA^	3.18 ± 0.80 ^cA^
Yellowness (b*)					
30	9.31 ± 2.05 ^aA^	7.04 ± 0.49 ^aA^	6.66 ± 0.58 ^aA^	5.19 ± 1.22 ^aA^	7.07 ± 0.59 ^aA^
60	6.26 ± 1.03 ^abB^	7.12 ± 0.98 ^abA^	6.86 ± 0.72 ^abA^	5.55 ± 1.28 ^aA^	7.84 ± 0.37 ^bA^
90	5.04 ± 0.18 ^aB^	5.16 ± 1.71 ^abA^	8.11 ± 1.10 ^cB^	5.83 ± 0.42 ^aA^	7.83 ± 1.17 ^bcA^

^A, B^—values marked with different capital letters are significantly different (*p* < 0.05) in the same sample; ^a–c^—mean values marked with different lowercase letters are significantly different (*p* < 0.05) at the same time. The results are presented as means ± standard deviation. C—control sample with sodium nitrite, S1—sample with acid whey and 2.8% NaCl, S2—sample with acid whey, 2.8% NaCl, and 0.05% ascorbic acid, S3—sample with acid whey and 2.8% NaCl:KCl (7:3), S4—sample with acid whey, 2.8% NaCl:KCl (7:3), and 0.05% ascorbic acid.

**Table 4:** Microbiological quality of fermented deer sausages after fermentation (30 days).

Sample	CB log CFU/g	TVC log CFU/g	LAB log CFU/g
C	1.45 ± 0.10 ^a^	3.84 ± 0.09 ^a^	3.36 ± 0.06 ^a^
S1	1.28 ± 0.06 ^a^	3.42 ± 0.30 ^b^	3.53 ± 0.07 ^abc^
S2	1.34 ± 0.09 ^a^	3.10 ± 0.07 ^b^	3.47 ± 0.13 ^ac^
S3	1.40 ± 0.09 ^a^	3.33 ± 0.26 ^b^	3.67 ± 0.16 ^b^
S4	1.35 ± 0.18 ^a^	3.13 ± 0.14 ^b^	3.63 ± 0.05 ^bc^

^a–c^—values marked with different lowercase letters are significantly different (*p* < 0.05) among the samples. C—control sample with sodium nitrite, S1—sample with acid whey and 2.8% NaCl, S2—sample with acid whey, 2.8% NaCl, and 0.05% ascorbic acid, S3—sample with acid whey and 2.8% NaCl:KCl (7:3), S4—sample with acid whey, 2.8% NaCl:KCl (7:3), and 0.05% ascorbic acid. CB—coliform bacteria; TVC—total viable count; LAB—lactic acid bacteria.

**Table 5:** Microbiological quality of fermented deer sausages after 90 days of production.

Sample	CB log CFU/g	TVC log CFU/g	LAB log CFU/g
C	2.38 ± 0.04 ^a^	3.50 ± 0.13 ^a^	3.39 ± 0.19 ^a^
S1	2.17 ± 0.28 ^a^	3.36 ± 0.47 ^a^	3.40 ± 0.20 ^a^
S2	2.10 ± 0.20 ^a^	3.55 ± 0.16 ^a^	3.54 ± 0.25 ^ab^
S3	1.66 ± 0.36 ^b^	3.59 ± 0.14 ^a^	3.46 ± 0.22 ^a^
S4	2.26 ± 0.13 ^a^	3.53 ± 0.05 ^a^	3.81 ± 0.09 ^b^

^a, b^—values marked with different lowercase letters are significantly different (*p* < 0.05) among the samples. C—control sample with sodium nitrite, S1—sample with acid whey and 2.8% NaCl, S2—sample with acid whey, 2.8% NaCl, and 0.05% ascorbic acid, S3—sample with acid whey and 2.8% NaCl:KCl (7:3), S4—sample with acid whey, 2.8% NaCl:KCl (7:3), and 0.05% ascorbic acid. CB—coliform bacteria; TVC—total viable count; LAB—lactic acid bacteria.

**Table 6:** The influence of applied technological treatments (variant) on the analyzed quality parameters based on the *p*-value obtained in ANOVA analysis.

	a_w_	pH	ORP	TBARS	L*	a*	b*	CB	TVC	LAB
Absolute term	*p* < 0.001									
Variant	*p* < 0.001	0.03	0.97	*p* < 0.001	0.80	*p* < 0.001	0.28	0.91	0.61	0.11

## Data Availability

The original contributions presented in the study are included in the article, further inquiries can be directed to the corresponding author.
